# Gendered factors for heated tobacco product use: Focus group interviews with Korean adults

**DOI:** 10.18332/tid/120103

**Published:** 2020-05-14

**Authors:** Kwanwook Kim, Jinyoung Kim, Hong-Jun Cho

**Affiliations:** 1 Department of Anthropology, College of Social Sciences, Seoul National University, Seoul, Republic of Korea; 2 Southern Gyeonggi Regional Smoking Cessation Center, Hallym University, Anyang, Republic of Korea; 3 Department of Family Medicine, Asan Medical Center, University of Ulsan College of Medicine, Seoul, Republic of Korea

**Keywords:** tobacco product, gender, focus groups, smoking cessation

## Abstract

**INTRODUCTION:**

Since June 2017, heated tobacco products (HTPs) have been on sale in Korea, comprising approximately 11.8% of total tobacco sales in April 2019. This research illuminates hitherto unexplored gendered factors influencing the use of HTPs.

**METHODS:**

The participants for the focus group interviews (FGI) were recruited among those who use or have used HTPs. Participants were separated into six groups (a total of 38 persons: 20 men and 18 women). Each FGI, lasting for two hours, was audio-recorded and transcribed, and subsequently coded to conduct a content analysis using NVivo V12.

**RESULTS:**

Both male and female participants shared the same opinion that HTPs were ‘less smelly’ and that despite their significant merit, HTPs had slightly different usages and places of use. First, male participants used them to avoid family members’ pressure to quit smoking, and female participants used them to avoid the stigma associated with female smoking. Second, men tended to use HTPs indoors, mostly in non-smoking areas, while women used them outdoors, mainly in the streets. Both genders were dissatisfied with the taste of HTPs and often used them in combination with combustible cigarettes (CCs). In terms of taste, dual use, absence of smoking cessation, and perception of harm, no definite gendered difference was found. Almost half of the participants considered HTPs to be less harmful than cigarettes, while others contended that they were equally harmful. Many agreed that there was no strong correlation between the use of HTPs and smoking cessation.

**CONCLUSIONS:**

Since HTPs have the potential to weaken motivating factors for smoking cessation in both male and female users, an understanding of their characteristics with gendered factors is beneficial to establishing policies to prevent the spread of HTP use and increase the overall rate of smoking cessation.

## INTRODUCTION

Since June 2017, heated tobacco products (HTPs) have been on sale in Korea, beginning with the ‘IQOS’ product of Philip Morris International (PMI)^1^. Korea Tomorrow & Global (KT & G) and British American Tobacco (BAT), the two other largest tobacco companies in Korea, likewise released ‘lil’ and ‘Glo’ products, respectively. One year after the launch of HTPs in Korea, the rate of use in 2018 was 2.13%; 4.01% for men and 0.24% for women. That is much lower than the general tobacco use rate (21.56%) but strongly suggests that 96.25% of HTP users are or were dual users^2^. It is also noteworthy that the rate of use amongst adolescents reached 2.8% one year after their introduction^3^. As of April 2019, sales of HTPs reached approximately 11.8% of total tobacco sales in Korea^4^. Given this situation, it can be said that Korea serves as a useful testbed for the market of HTPs.

In the late 1980s, Korea became an attractive market for multinational tobacco companies, due to the high smoking rates in males (71% in 1985), and low female smoking rates (8% in 1985) with gradual cultural changes, particularly regarding the nationalistic attitudes toward imported tobacco brands^5^. Korea has made significant headway in attempts to curb smoking rates by introducing an 80% tax increase on tobacco products in 2015. However, as in the situation 30 years ago, Korea is now becoming an international strategic target for HTP sales as a result of high male smoking rates (38.1% in 2017) and relatively low female smoking rates (6% in 2017)^5–7^. Tobacco companies have traditionally marketed using gendered perspectives (i.e. in a way that emphasises femininity and masculinity) systematically and continuously^8^. In this reality, Amos et al.^9^ strongly suggested that a gendered perspective should be seriously taken into account in both research and the formulation of tobacco policies, especially in regard to girls’ and women’s smoking^9^. Recently, however, multinational tobacco companies have begun to recruit existing smokers, female non-smokers and youth with a so-called hybrid cigarette equipped with smell reduction. These state-of-the-art tobacco products have the risky potential to appeal to female Korean smokers who have had to devise a smoking strategy to avoid social stigma^10^. Thus, it is necessary that tobacco research and policies include a gendered perspective in line with the introduction of new tobacco products.

Until recently, research on HTPs has been conducted in two broad categories. One strand constitutes the nicotine components used, harmful ingredients contained, and the biochemical effects HTPs have on animals and humans, such as the IQOS aerosol’s effects of impairing arterial function^11–14^. The other strand concerns the prevalence and perceptions of HTPs in Japan and Italy, countries where the product was first marketed in 2014^15-17^. In 2016, comparative research conducted in Japan and Switzerland showed differences between European and Asian smokers in accepting HTPs.^18^ Both of these studies show cultural differences in the use of HTPs between countries. However, they did not account for any gendered factors affecting use within a country. As with the marketing plans of tobacco companies, knowledge of these gendered factors can be beneficial to implement a country’s anti-smoking policy.

This study does not plan to reaffirm the results of previous research on HTPs, which are mainly quantitative studies. It attempts to further secure underlying data for developing gender-sensitive anti-smoking strategies. Although focus group interview (FGI) methodology may provide limited information, it is a method that can be used in the timely development of appropriate tobacco policies. The present study aims to investigate reasons for and patterns of gendered differences in HTP use.

## METHODS

### Sample and recruitment

This study conducted focus group interviews (FGIs) to elicit and compare the differences of the HTP smoking experience between males and females. The participants were users of HTPs, both current and former, who were divided into six groups (with a total of 38 persons: 20 men and 18 women). We aimed to recruit users of representative HTP brands in Korea (e.g. IQOS, Glo, lil). We also included both single and married participants, with or without children, to observe any potential influence that family dynamics had with regards to smoking. Males and females of legal age to smoke (aged ≥19 years) were recruited, and classified into four groups: G1, males, aged 19–34 years; G2, males, aged 35–49 years; G3, females, aged 19–34 years; and G4, females, aged 35–49 years. To ensure sufficient representation of dual or triple users after the FGI in the original four groups, two further groups of male and female users (G5 and G6) were recruited (Supplementary file, Table 1). The recruitment process was conducted through EMBRAIN, a Seoul-based research firm (http://www.embrain.com/eng/). To protect the confidentiality of the participants, we received their consent for collecting and using personal information. This process was approved in advance by the Institute Review Board of Asan Medical Centre (2018-1180).

**Table 1:** Comparison of the ‘coverage’ of the main codes by Nvivo12

*Groups*	*Coverage, the percentage (%) of the area occupied by the specific code*				
	*Smell talk*	*Comparative experience of EC*	*Convenience*	*Drawbacks of taste and smoke volume*	*Recognition of gender differences in HTP use*
**G1** Male <35 years (N=8)	4.75	5.48	0.99	3.62	1.48
**G2** Male ≥35 years (N=6)	5.57	3.96	2.67	2.52	0
**G3** Female <35 years (N=6)	6.96	2.21	7.57	3.39	6.97
**G4** Female ≥35 years (N=6)	11.94	2.05	4.83	2.14	3.48

### Data collection and analysis

Subjects met in EMBRAIN’s Seoul FGI room. Group interviews with groups G1 to G4 were conducted between 15 and 19 October 2018, while G5 and G6 were interviewed between 9 and 20 February 2019. One of the authors (KKW) took on the role of a moderator. Each FGI lasted two hours, based on an interview schedule with semi-structured questions. The main questions in the schedule covered: 1) types of HTPs used and the reason for that choice; 2) locations where HTPs were frequently used and any changes after use of HTP; 3) tobacco use patterns (single or dual/triple use, change in smoking frequency and smoking cessation plan); and 4) product satisfaction and understanding about its harmfulness, secondhand effects and any contributions it had made to smoking cessation. Before commencing the group interview, each participant completed a self-administered questionnaire (population sociological characteristics, smoking history etc.), a flowchart of the patterns of their last two years of tobacco use, and a current daily tobacco use schedule. All FGIs were audio-recorded and transcribed. We used NVivo V.12 to code six transcriptions and checked the most frequent codes to compare differences between male and female participants^19,20^. To conduct content analysis^21^, one author (KK) performed open coding, grouped codes, and created categories based on the aforementioned four main questions. After two authors (JK and HJC) examined those results, three authors analysed the findings. We also used the contents of the self-completed smoking pattern flowchart to understand the reason for changes in smoking behaviours.

## RESULTS

### General characteristics

In total, 687 codes were generated and categorised into four main themes: 1) reason and purpose of HTP use (code=282); 2) place of HTP use (code=97); 3) ways of HTP use (code=77); and 4) perceptions of harmfulness and smoking cessation effects of HTPs (code=231). The code with the highest frequency was the smell of HTPs (n=56), specifically their reduced smell and unique odour. Next, in descending order, were their *characteristics compared to liquid type electronic cigarettes* (n=30), *convenience in use* (n=24), *negative opinions on the value of HTP as alternative smoking cessation aid* (n=22), *and negative opinions as an aid for stopping smoking* (n=19). Table 1 summarises *coverage*, the percentage of the area occupied by the specific code among the entire recorded contents of the relevant group. This metric, calculated by NVivo from the main codes of groups 1–4, can help us to understand the relative gender and age differences in the subject of interest, based on the assumption that people talk more about issues that are important to them.

According to Table 1, female and older participants spoke more about HTPs’ smell; this was especially noteworthy for females aged >35 years (mainly those married with children). Young male participants spoke more about their experience with EC compared to females. Females, mainly younger (and employed), talked more about the various convenience aspects of using HTPs. There was little difference in gender regarding the drawbacks of lack of taste and smoke volume of HTPs. Females (especially the younger group) acknowledged more readily than did males the fact that differences exist between male and female smokers in the reasons for using HTP.

### Reason and purpose of HTP use

As has been well-established by previous studies, the smell of cigarettes was the main reason for using HTP for both male and female users. Nevertheless, there was a gender difference in the cause for concern about the smell of cigarettes, especially regarding the person(s) to whom participants thought the smell was an issue. Males tended to identify smell as a problem when it came to their familial responsibilities with their wives and children. Many participants felt guilty for using cigarettes due to their harmfulness to health and exposing their family members to secondhand smoke. For these participants, these feelings, usually recognised, and revived by the very smell of tobacco, could be reduced through the use of HTPs with a relatively low odour. Therefore, males were more concerned with the ‘physical’ characteristics of the cigarette smell as a reminder of the harmfulness of tobacco.

*‘When I got married, my wife knew that I was a smoker and did not care too much. But when she got pregnant, she kept telling me, “Your body smells of cigarettes. Don’t come near, it’s bad for a child”. She told me a lot to stop smoking. So, I thought about various ways, and finally bought IQOS which was easily available. I think it would be better to choose one that doesn’t smell to my family.’* (Male, 39 years)

Unlike the male participants, female participants were more interested in the ‘socio-cultural’ rather than physical characteristics of the tobacco smell. In other words, women were conscious that their tobacco smell would expose their smoking habit in a patriarchal society where female smoking is still a highly stigmatised activity, particularly in the workplace. Therefore, unlike men, they were reluctant to disclose their smoking habit. In particular, women with children were extremely vigilant about concealing such socially unacceptable behaviour as smoking, when among other parents and their children’s teachers, because of the perception that they should be a morally upright ‘agi-eomma’ (a baby’s mother). For these reasons, women chose HTPs to maintain their social status as a righteous working woman or mother.

*‘I should not let my colleagues in the company notice the smell from my smoking. Since my sister introduced me to “lil”, I have used it while working and smoked CCs at home.*’ (Female, 22 years)

*‘For me, [the] IQOS has solved every interpersonal problem caused by the smelly cigarette. Now I have been able to avoid uncomfortable gazes, [I am] liberated from the smell, and [I have] improved interpersonal relationships with my children’s teachers or other parents. I used to be unable to smoke openly because I was given kind of a name tag called “agi-eomma” (a baby’s mother).’* (Female, 42 years)

In addition, several male participants mentioned health-related reasons for the use of HTPs, such as reduction of sputum or tartar. On the other hand, some female participants mentioned the design of the IQOS as the reason for choice.

### Place and ways of HTP use

Male, female, current and former users of HTPs reported using them indoors (e.g. in the office, bedroom, toilet, living room, balcony etc.) in places where they have seldom attempted smoking before, as well as public spaces (hospital, hotel, theatre, public restroom, street etc.) where smoking is illegal. However, the gender difference here is that males apparently tend to use HTPs more indoors (e.g. toilet), whereas women spoke more about their use of HTPs outdoors (e.g. street).

*‘I smoke HTP while taking a dump in the toilet. But I am not an exception. Everyone does the same. It is a kind of habit. It has come back again. Back in the days when I was a university student, I used to smoke while going to the toilet. Since then, the activity was legally forbidden. HTP helped me revive my forgotten habit.’* (Male, 28 years)

*‘I’ve never smoked indoors but what I feel like a whole new world with [the] IQOS is so-called “street smoking”.’* (Female, 34 years)

*‘Generally, particularly women can’t smoke while walking on the street. But, HTP is less smelly and less smoky. So now, I enjoy it on the street.’* (Female, 37 years)

Meanwhile, in terms of ways of using HTPs, both male and female users pointed out that HTPs generally lack a typical tobacco flavour and the ‘throat hit’ feeling of a CC. One female user compared CCs to ‘charcoal-grilled meat’ and HTPs to ‘steamed meat’ to illustrate the distinct taste difference. Therefore, many of the participants were compensating for these deficiencies (e.g. the ‘steamed’ taste) by using both CCs and HTPs or intermittent use of CCs. Some of the participants from both male and female users preferred to use only HTPs due to feeling disgusted now with the smell of the original CCs. Except in these cases, despite being dissatisfied with the shortcomings of HTPs, the typical trajectory of male and female users consisted of dual use of CCs and HTPs for a period, followed by a gradual return to smoking CCs only. Although there were two users (one male G1, one female G3) who reported quitting smoking after using HTPs, this was largely due to strong recommendations and support from people around them (family members, partners etc.) or apparent personal health problems (e.g. asthma, chronic tonsillitis, and vascular disease).

With such distinct advantages and disadvantages of HTPs, the users adapted to each alternative. In particular, in terms of dual and triple users in focus groups G5 and G6, male and female users both had perspectives on the advantages and disadvantages of three types of tobacco products (CC, EC and HTP) with five factors at the core — smell, taste, convenience in use, health problems, and cost aspects (Supplementary file, Tables 2 and 3). While both male and female smokers, as mentioned earlier, made frequent reference to smell of HTP, men tended to emphasise contrast mainly in the taste of tobacco among the three types. Women were more concerned about the convenience in use (e.g. situational convenience, convenience of smoking location, and device operation).

### Perceptions of harmfulness and smoking cessation effect of HTP

In this FGI research, no differences were found between male and female users with respect to the perception of harmfulness and smoking cessation effect of HTPs. Almost half of the respondents thought that the harmfulness of HTPs was no different from that of CCs, and about half of them thought that HTPs were in fact beneficial by comparison. Participants’ beliefs that HTPs were less harmful to the body were based on their own experiences, such as reduction of sputum and tartar. In contrast, participants who judged HTPs to be just as harmful as CCs relied on the fact that sticks of HTPs are derived from the same tobacco leaf as CCs.

*‘First of all, now I don’t have a cough and sputum, and I feel better when I wake up in the morning. In the past, if I stopped smoking CCs for three days and then smoked again, I felt dizzy and sat down. But, in the case of HTP, I don’t feel dizzy at all. So, I guess it’s a little better.’* (Male, 31 years)

*‘I actually broke the HTP stick and saw the contents inside. I could see the same tobacco leaves of those of CCs.’* (Male, 26 years)

Although there were some differences in opinion regarding the harmful effects of HTPs, male and female participants were almost identical in their perspectives on their value for encouraging smoking cessation. They rarely recognised them as a supplement for use in the smoking cessation process and did not believe HTPs had a positive effect on contemplation of smoking cessation. Instead, there was a tendency to accept them merely as another ‘type’ of cigarette, albeit with a weaker smell, and some participants even believed that the HTPs would be more difficult to stop using since they were designed to complement the significant shortcomings of CCs. In some cases, in fact, HTP users became less interested to stop smoking since HTP use helped them avoid the pressure to stop smoking from the non-smokers around them.

*‘I think one should stop smoking immediately if he wants to quit smoking. You should not even use HTP. HTP complemented the weaknesses of CCs and made them their [the HTP] advantages. So, it seems to be more challenging to quit HTPs than CCs.’* (Male, 28 years)

*‘I think that the HTP only has a difference in whether it is lit or not. So, I don’t think it logical that a cigarette, even if it is not lit, can be used for a smoking cessation aid.’* (Female, 36 years)

## DISCUSSION

The most well-known characteristic feature of HTPs is that they are ‘less smelly’, according to results from Korean research and foreign studies^1,2,18^. Since the launch of HTPs, although basic research on the rates of HTP use and cigarette use patterns (e.g. dual or triple use) has been conducted, differences in users’ specific experiences have not yet been scrutinised^2^. Reaffirming that ‘smell’ is the most significant factor in the use of HTPs, this study also showed that there are gender differences for ‘less smelly’ (Table 1). Analysing the detailed interview data, men and women have the following differences concerning features. For males, use of HTPs frees them from accusations of harming others with secondhand smoke by reducing visible smoke. For females, it makes daily life more convenient as it enables them to hide their smoking, thereby indirectly avoiding the gender norm of non-smoking imposed on women. Thus, in terms of tobacco policies: 1) for men, the characteristic of less tobacco odour tends to remove external factors that lead to quitting smoking (e.g. family members’ pressure to quit smoking); 2) for women, it also helps to break social barriers (e.g. stigmatised views towards female smoking) that prevent them from trying or continuing the habit of smoking. Therefore, it can be seen that the ‘less smelly’ feature of HTPs hinders smoking cessation in different ways for male and female users.

The reason for paying attention to the gender differences concerning the ‘smell’ of HTPs can be illustrated by examining Indian research into smokeless tobacco use^22^. In India, even though various traditions are not in favour of female smoking, there is no taboo or stigma against using ‘smokeless’ tobacco. Therefore, most female tobacco users prefer to use smokeless products. Smokeless tobacco, which is also odourless, is not considered harmful. Instead, it is often found to be a traditional medicine, despite the fact that its use among pregnant women increases rates of stillbirth and low birthweight infants by 2–3 times. There is also an increased incidence of oral and esophageal cancer in those who use smokeless tobacco products^22^. The lessons for tobacco policy here are as follows: if there is no visible smoke and no direct or indirect damage and discomfort to others, it allows the user (particularly females) to escape the strict gender norms of smoking. It could be regarded as freedom or human rights for the individual^9^, but this, in turn, can cause health damage to both women and the fetus. Therefore, it is essential to note the ‘less smelly’ feature of HTPs in Korea when it comes to devising policy.

Furthermore, given that female smoking rates in Korea are under-reported due to the prevailing negative societal view^23^, the fact that HTPs help conceal and sustain women’s smoking could be another barrier in the promotion of gender-sensitive tobacco policies. In Korea, marital status could play an important role in protecting smoking in women compared to men^24^. Thus, HTPs may also be an obstacle when it comes to developing health policy around tobacco use, considering the excerpt from an interview in the present study in which the use of HTPs helps ‘a baby’s mother’ hide her smoking habit.

Next to the ‘less smelly’ advantage of HTP, it frees both male and female users from the restrictions on smoking places. Men have revisited smoking areas (toilets, indoors etc.) that were previously made unavailable due to the expansion of strict smoking cessation policies. For women, it was more convenient to smoke in places, such as in the street, that were previously made out-of-bounds by ‘social gaze’ rather than in spaces made off-limits by policy. Thus, the introduction of HTPs could potentially dilute or cancel out the contribution of smoking place control to smoking cessation made by anti-smoking policies or societal norms. In terms of smoking cessation policy, it can be emphasised that, for men, this goes beyond simple personal deviation and instead moves against the overall indirect smoking ban. Furthermore, for women, freedom from immediate restrictions is not in any way an ‘expansion of actual human rights’.

Most male and female participants used HTPs in combination with CCs because of the perceived lack of taste in HTPs. Although the duration of HTP use differs depending on gender and individual differences, it should be noted that when they stopped using HTPs, the return to smoking CCs was more common than complete cessation of smoking. In other words, there was no strong correlation between the use of HTPs and smoking cessation. Almost all the male and female participants who were interviewed agreed with this perspective. As highlighted in previous quantitative research on the use of HTPs in Korea, HTPs are not a substitute for CCs, but are rather complementary products^2^. Meanwhile, in the cases of poly-users with HTPs, the gender difference is noteworthy when establishing a smoking cessation policy; men focused on comparing the taste of various tobacco products, while women focused more on comparing whether they were convenient to use. Thus, when conducting smoking cessation education for male HTP users, it is important to emphasise that the taste of tobacco and its capacity for harm are entirely different (e.g. there is no proof that a milder taste made it less harmful). For female users, it is recommended that policy emphasises that the convenience of tobacco use can lead to increased exposure to hazardous substances.

Finally, there were no definite differences between male and female users in their perceptions of the harm posed by HTP use. Almost half of the participants said that based on personal experience (reduction of coughing, sputum, tartar etc.), smoking heated cigarettes was less harmful. In addition, others disagreed with them and perceived that HTPs were as harmful as cigarettes but with less odor. In other words, it can be helpful to stress that HTPs may have short-term physical benefits for users, but the likelihood of exposure to harmful substances may be high because of possible long-term use.

### Limitations

This study has several limitations. First, the sample size is small (20 males and 18 females). Although the study is based on interviews, and limiting the number of people involved is unavoidable, the group interview results might be limited in that they do not represent all the experiences of HTPs. Second, the selection of participants was not random. Although the participants were recruited voluntarily from a pool of candidates who registered with a research firm, they were not randomly selected. Third, because one of the strengths and weaknesses of the focus group interview is that participants could freely speak to each other, there is a risk that the opinions of some users were overemphasised. Finally, while one researcher took on the role of a moderator during every group interview and tried to exclude his judgments as much as possible, there is the possibility that participants filtered their experience during discussions. Despite these limitations, research conducted using the FGI method is able to collect and compare individual users’ experiences, within short duration, in order to respond quickly to the expansion of HTPs and develop timely tobacco policies.

## CONCLUSIONS

This study examined gendered factors for HTP use in Korea through FGIs. As a result, men and women agreed that the most characteristic feature of HTPs is that it is ‘less smelly’. However, there were gendered differences in the ways of using the HTP and the choice of smoking place. There was no gendered difference in perceptions of the taste of HTPs, dual use of HTPs and other tobacco products, absence of the effect of smoking cessation, and perception of HTP-related harm. An understanding of these gendered factors—whether similarities or differences—can be beneficial for developing anti-smoking policies for HTPs. In particular, this study revealed that HTPs have the potential to weaken external (e.g. advice) and internal (e.g. contemplation) motivating factors for quitting smoking in both male and female users. These two aspects should be considered when formulating policies to prevent the spread of HTPs and increase the overall rate of smoking cessation.
