# Supplementary material for: Gendered factors for heated tobacco product use: Focus group interviews with Korean adults
Source: Tob Induc Dis. 2020 May 14;18:43. doi: 10.18332/tid/120103 (PMC7252430; doi:10.18332/tid/120103)
Supplement: Supplementary file 1 [file TID-18-43-s1.pdf]

Supplementary Material for the Article:

Gendered factors for heated tobacco product use: Focus group interviews with Korean adults **This material supplements, but does not replace, the peer-reviewed paper in *Tobacco Induced Diseases***

Supplementary Table 1. Characteristics of participants from each group in terms of tobacco use patterns

Groups	Current tobacco use patterns						Total number
	None	CC*	EC*	HTPs (I,G,L)*	Dual users	Triple users	
G1 (male, <35 years old)	1 †	2	0	3 (I, G, L)	2 (CC+G)	0	8
G2 (male, ≥35 years old)	1†	1	0	3 (I, G, L)	1 (CC+EC)	0	6
G3 (female, <35 years old)	1†	1	1	1 (I)	2 (CC+L/G)	0	6
G4 (female, ≥35 years old)	0	2	0	2 (I)	2 (CC+I)	0	6

G5 (male, <50 years old)	0	0	0	0	6(1=CC+EC/5=CC+HTP(I=3, I&L=1, I&G=1))	0	6
G6 (female, <50 years old)	0	0	0	0	5(2=CC+EC/3=CC+HTP(1=I, 1=G, 1=L))	1(CC+EC+I)	6
	*CC = Combustible cigarette, EC = Electronic cigarette, HTP = Heated Tobacco Product<br>*I = IQOS, G=Glo, L=lil<br>† G1 non-smoker = quit smoking 1 year ago after IQOS use, G2 non-smoker = quit smoking 9 months ago due to severe neck pain from cervical disc herniation, G3 non-smoker = quit smoking 10 months ago after IQOS use.						

Supplementary Table 2. Comparison of advantages and disadvantages of the three types of tobacco products in Group 5 (male)

**Types of tobacco products**	**Five main factors to compare among male dual or triple users**				
	Smell	Taste	Convenience	Health problems	Cost aspects
**CC**	Smell worst	best, incomparable but the burnt taste is strong	Uncomfortable with cigarette ash in a pocket	Tartar, sputum	
**EC**	Very little smell enough to cause excessive vaping indoors unwittingly	Taste not good enough, overcome it with aromatic liquid like menthol	A little uncomfortable, but good to vape for a long time, addictive though	Frequent gum problems	Reasonable price of liquid nicotine
**HTP**	Smell awful, uncomfortable (like the smell of steamed corn, etc.)	Good not having the burnt taste, but using aromatic stick to avoid uncomfortable smell	Inconvenient for continuous smoking	No tartar	A little expensive

Supplementary Table 3. Comparison of advantages and disadvantages of the three types of tobacco products in Group 6 (female)

**Types of tobacco products**	**Five main factors to compare among female dual or triple users**				
	Smell	Taste	Convenience	Health problems	Cost aspects
**CC**	Smells so bad when smoking indoors	Bad smell but, excellent taste	Hard to clean up after smoking secretly, stressful from hiding	Tartar, stomach-ache	
**EC**	Very little smell, then convenient to vape with drinking or indoors	A variety of aromatic liquids available	Difficult in operation, leakage is a problem with nicotine liquid, concern with safety of the devices, easy for f continuous use resulting in excessive vaping	Immediate gum pain, risk of excessive continuous use	EC choice due to increase in the CC price

**HTP**	Weak enough to smoke indoors, but bothersome with characteristic smells like fish, steamed mugwort, puppy hair, and fart	Unique smells make taste bad	Experience a lot of device-related problems; easily broken iron core, inconvenient for cleaning sticky residue, many minor defects, and very inconvenient service centre	No tartar	

© 2020 Kim K. et al.
